# The Protective Effects of Salubrinal on the Cartilage and Subchondral Bone of the Temporomandibular Joint under Various Compressive Mechanical Stimulations

**DOI:** 10.1371/journal.pone.0155514

**Published:** 2016-05-19

**Authors:** Juan Wen, Yuanyuan Jiang, Caixia Zhang, Sheng Chen, Huang Li

**Affiliations:** 1 Orthodontic Department, Nanjing Stomatological Hospital, Medical School of Nanjing University, Nanjing, Jiangsu, People's Republic of China; 2 Pathological Department, Nanjing Stomatological Hospital, Medical School of Nanjing University, Nanjing, Jiangsu, People's Republic of China; University of Akron, UNITED STATES

## Abstract

Excessive mechanical loads on the temporomandibular joint (TMJ) can cause mandibular cartilage degradation and subchondral bone erosion, but the treatment of these conditions remains challenging. Salubrinal, which target eukaryotic translation initiation factor 2 alpha, has been shown to have multiple beneficial effects on skeletal tissue. Here, we examined the effect of a Salubrinal injection on the mandibular cartilage and subchondral bone of the TMJ under various compressive stresses. We conducted *in vivo* analyses in rat models using various compressive stresses (40 g and 80 g), and we observed time-related degeneration and pathological changes in the cartilage and subchondral bone of the TMJ at days 1, 3 and 7 through histological measurements, subcellular observation, and changes in proliferation and apoptosis. After the Salubrinal injection, the thickness of the cartilage recovered, and the pathological change was alleviated. In the Salubrinal/light (Sal/light) compressive stress group, the drug altered the proliferation and apoptosis of chondrocytes most significantly at day 1. In the Salubrinal/heavy (Sal/heavy) compressive stress group, the drug increased the proliferation of chondrocytes most significantly at day 1 and reduced the apoptosis of chondrocytes most significantly at day 7. Salubrinal also increased the area of the bone trabeculae and suppressed inflammatory responses and pathological change in the subchondral bone of the TMJ. Together, these results indicate that the administration of Salubrinal reduces apoptosis and strengthens the proliferation of chondrocyte to varying degrees at days 1, 3 and 7 under various compressive mechanical stresses, both of which contribute to the recovery of cartilage thickness and the alleviation of pathological change. Salubrinal also suppresses inflammatory responses and pathological change in the subchondral bone of the TMJ.

## Introduction

The temporomandibular joint (TMJ) is a diarthrodial joint in the human body. Appropriate mechanical loads on the TMJ are vital for the maintenance and remodeling of mandibular condylar cartilage. However, excessive mechanical stress, which can be generated by parafunction, has been recognized as a major factor in the development of osteochondral damage or diseases of the TMJ [1]. Previous studies have reported increased apoptosis of chondrocytes in cartilage and reduced proliferation of mandibular chondrocytes *in vivo* and *vitro* in response to excessive mechanical stress [2–4]. However, the treatment of osteochondral damage and TMJ diseases still remains a major challenge.

We have proven that endoplasmic reticulum stress (ERS) regulates mechanical stress-induced cartilage thinning [3]. Salubrinal, which is an inhibitor of ERS, can elevate the phosphorylation of eukaryotic translation initiation factor 2 alpha (eIF2α) [5]. Through eIF2α-mediated transcriptional and translational regulation, Salubrinal has been shown to have multiple beneficial effects on skeletal tissue. First, Salubrinal downregulates the expression and activity of Matrix Metallopeptidase 13 (MMP13), in chondrocytes, suggesting its potential use for protecting chondrocytes and treating degenerative diseases, such as osteoarthritis [6]. Second, the elevated phosphorylation of eIF2α induced by Salubrinal could stimulate osteoblastogenesis and bone formation [7]. Third, Salubrinal suppresses osteoclastogenesis, followed by bone resorption and protection [8,9].

Given the important role of Salubrinal in cartilage and bone, we hypothesized about the potential effects of Salubrinal on the cartilage and subchondral bone of the TMJ under various compressive mechanical stresses. In the current study, we explored the effects of different compressive mechanical stresses on the cartilage and subchondral bone of the TMJ at different times after injecting Salubrinal. We conducted *in vivo* analyses using light and heavy compressive stresses rat models, in which we observed Salubrinal's effects on the cartilage and subchondral bone of the TMJ at days 1, 3 and 7. We used hematoxylin and eosin (H&E) staining and transmission electron microscopy (TEM) to observe the morphology of cartilage, chondrocytes and subchondral bone. The thickness of the cartilage, the number of chondrocytes, and the area of subchondral bone were also evaluated. We detected the proliferation and apoptosis of chondrocytes under compressive stresses and after injecting Salubrinal.

## Materials and Methods

### 2.1 Animals

A total of 75 male Sprague-Dawley (SD) rats (provided by Nanjing Medical University) that were 6 weeks old and weighed 200±20 g were used in this study. All rats were acclimatized to the laboratory conditions with food and water available 1 week before the experiment. The rats were randomly divided into five groups: a light compressive mechanical stress group (40 g), a heavy compressive mechanical stress group (80 g), a Salubrinal/light compressive mechanical stress group, a Salubrinal/heavy compressive mechanical stress group, and a control group (n = 15). In each group, rats were randomly selected for the experiment to exclude selection bias. The animals were housed in a light- and temperature-controlled room and given unrestricted access to food and water during the experimental period.

Compressive mechanical stresses were loaded onto the TMJ as previously described [3]. A rubber band was tied between the jig and the anchorage hooks to load force on both sides. Rats in each subgroup wore the appliance for 1, 3, or 7 days with gender- and age-matched controls. The care and use of the animals in this study were undertaken in compliance with the guidelines of and permission from the Animal Care Committee of Nanjing University.

### 2.2. Salubrinal injection in TMJ

Salubrinal (Sal, Calbiochem, Germany) was dissolved in dimethylsulfoxide and further diluted with saline. The anesthetized rats were injected with Salubrinal [10,11] (15 μl, 75-μM solution) in the articular capsules of the condyle [12] 30 minutes before compressive mechanical stress loading. A tailored microinjector was inserted just below the zygomatic arch between the eye and ear until the outer surface of the mandibular ramus was reached. The orientation of the needle head was adjusted to enable the needle to pass along the bone wall and finally reach the TMJ region. The injections were repeated every other day.

### 2.3 Sample preparation and histological measurements

Rats were sacrificed by cervical dislocation under anesthesia. The TMJ was removed and fixed in 4% paraformaldehyde for 24 hours. The specimens were decalcified in EDTA solution for 8 weeks and embedded in paraffin after thorough rinsing. Blocks were cut into sagittal sections with a uniform thickness of 4 μm. The histological changes in the mandibular cartilage were examined by H&E staining. The other specimens were stained using other methods as follows. For the histological measurements, we drew one line from the attachment of the lateral pterygoid muscle to the attachment of bilaminar zone and two lines radially from the center mark of the first line to create three angles of 60° that divided the condyle into three parts: anterior, middle and posterior. We mainly focused on changes to the posterior part of the middle third (S1 Fig), which is the main load-bearing area based on the direction of force application [3]. The thicknesses of the cartilage and the number of chondrocyte cells were measured at the square located in the middle third of the cartilage (S1 Fig). In every region, the thickness was measured three times, and the mean of the three measurements was used for statistical analysis. The measurement of the square of the bone trabecula of the subchondral bone was obtained using the same procedure. In every region, the square was measured three times, and the mean value was used for the final results.

### 2.4 Transmission electron microscopy

Three tissue samples in each subgroup were selected for TEM investigations. Fresh small tissue blocks (1 mm^3^ volume) from the mandibular condylar cartilage were fixed in a solution containing 2.5% glutaraldehyde in 0.1 mol/L cacodylate buffer (pH 7.4) at room temperature. The specimens were decalcified in EDTA for 10 days, rinsed with phosphate buffered saline (PBS), and fixed in 2% osmium tetroxide (OsO4) in 0.1 mol/L cacodylate buffer (pH 7.4) for 2 hours. The material was then dehydrated through a graded series of ethanol and propylene oxide. Ultrathin sections (75-nm thick) were stained with uranyl acetate and lead citrate and examined using a Tecnai TEM (FEI, USA) operated at 80 kV.

### 2.5 Immunohistochemical staining

Changes in the expression of Ki67 were observed by immunochemistry. The sections were rinsed with PBS. The antigens were retrieved by heating in a pressure cooker, rinsed with PBS buffer, treated with 3% H_2_O_2_ in PBS, rinsed again with PBS, incubated with 10% normal rabbit or goat serum at room temperature for 10 minutes, and treated with Ki67 antibodies (1:100, Abcam, Hong Kong) in a moist chamber overnight at 4°C. After incubation with secondary IgG antibodies (HRP conjugated) for 30 minutes at 37°C, the sections were rinsed and incubated for 30 minutes with SP complex at 37°C. The samples were stained with diaminobenzidine (SP immunochemistry kit, Boster Biological Technology, Ltd., Wuhan, China). The cell nuclei were counterstained with hematoxylin. The stained samples were embedded on microscope slides with neutral resins. A color difference was used to delineate positive and negative areas, which were measured with Image-Pro Plus software.

### 2.6 TUNEL staining

Tissue sections (4-μm thick) were prepared according to standard protocols for H&E staining. The apoptotic cells in each section (3 sections per rat, 4 rats in each subgroup) were visualized using terminal deoxynucleotidyl transferase mediated d-UTP nick end labeling (TUNEL) according to the manufacturer's instructions (Boster Biological Technology, Ltd., Wuhan, China). Briefly, after deparaffinization and rehydration, sections were treated with proteinase K (Boster Biological Technology, Ltd., Wuhan, China) for 20 minutes at room temperature. After rinsing, they were incubated at 37°C for 60 minutes in the TUNEL reaction mixture and washed thoroughly in PBS. Slides were then mounted and observed under a fluorescence microscope (BX-60, Olympus, Tokyo, Japan) with appropriate filters (excitation 450–500 nm, emission 515–565 nm). Negative control slides were incubated with a labeling solution (without terminal transferase) instead of the TUNEL reaction mixture. Positive control slides were incubated with bovine pancreatic DNase I (Boster Biological Technology, Ltd., Wuhan, China, 0.01 mg/ml). The number of apoptotic cells was calculated with Image-Pro Plus 6.0 software.

### 2.7 Statistical analysis

All experiments were repeated 3 times. The data were expressed as the mean ± SD and compared using a multi-factor analysis of variance depending on whether they were normally distributed. All statistical analyses were performed using SPSS version 17.0 software, and P<0.05 was considered statistically significant.

## 3. Results

### 3.1 Experimental outcomes

No infections were detected at the injection sites or the force-loading sites during the experiments. We did not observe any abnormal behavior or diminished food intake. All devices were found to be intact and in place at the time of sacrifice.

### 3.2 Recovery of degeneration and pathological changes of the mandibular cartilage under various compressive stresses after injecting Salubrinal

**Fig 1:**
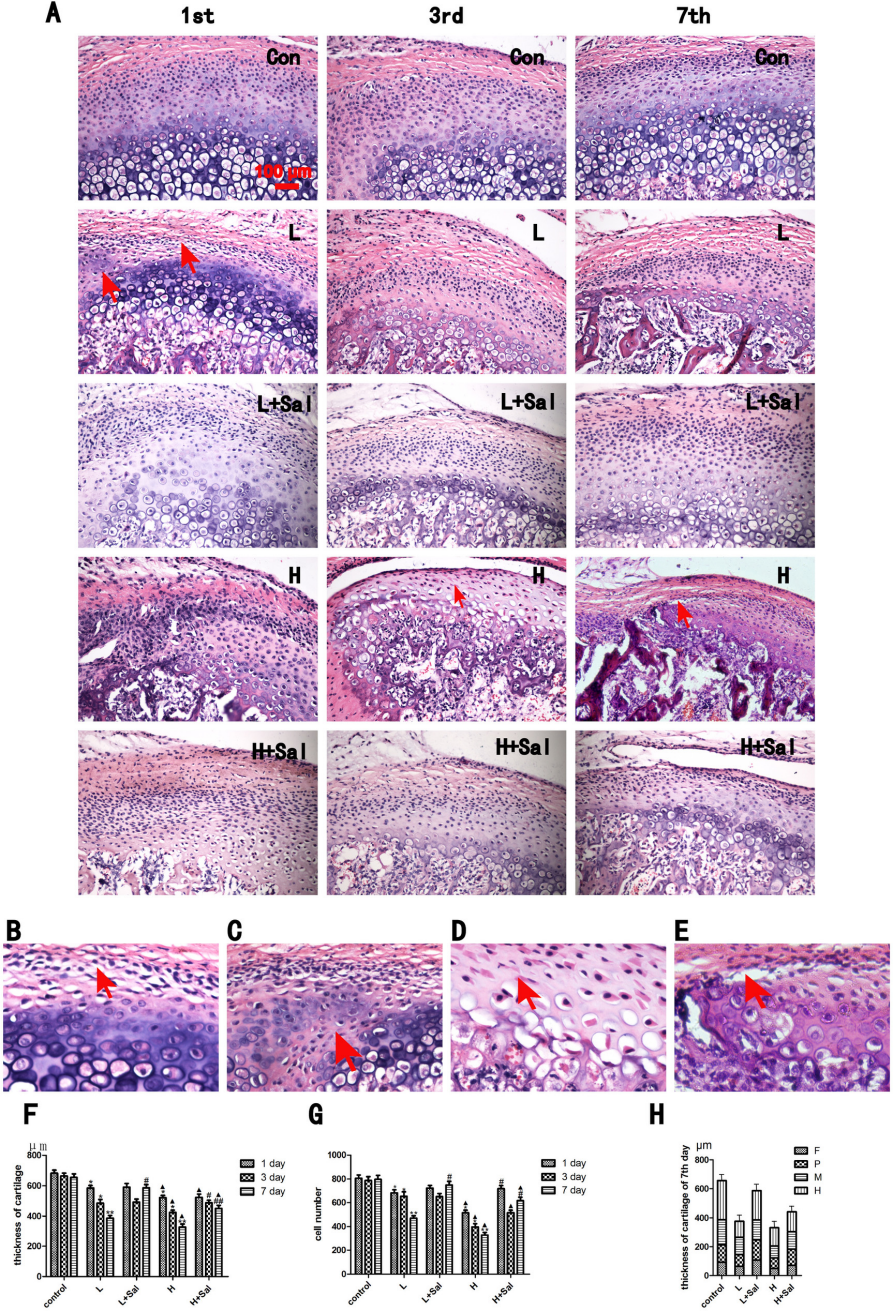
Histological examinations of cartilage under various compressive stresses and after injecting Salubrinal. (A) The thickness change in the condylar cartilage under various compressive stresses after injecting Salubrinal at days 1, 3 and 7. (B) Higher magnification in the day-1 light compressive stress subgroup. More lacunas were located in the proliferative zone. (C) Higher magnification in the day-1 light compressive stress subgroup. Chondrocytes in the proliferative zone penetrated into the hypertrophic zones. (D) Higher magnification in the day-3 heavy compressive stress subgroup. The nuclei of chondrocytes in these degraded areas were typically pyknotic and homogeneous, and the cytoplasm appeared condensed and did not fill the lacuna. (E) Higher magnification in the day-7 heavy compressive stress subgroup. The chondrocytes in the mandibular cartilage appeared disordered and disarranged. (F) Quantitative analysis of cartilage thickness. (G) Quantitative analysis of the number of cells. (H) Quantitative analysis of cartilage histological layers at day 7. Con: control group; L: light compressive stress group (40 g); H: heavy compressive stress group (80 g); L+Sal: light compressive stress group with injected Salubrinal; H+Sal: heavy compressive stress group with injected Salubrinal; F: fibrous layer; P: proliferative layer; M: mature layer; H: hypertrophic layer. Scale bars, 100 μm. (* indicates the comparison between the compressive stress groups and the control group, * p<0.05, ** p<0.01; ▲ indicates the comparison between light and heavy compressive stress groups, ▲ p<0.05, ▲▲ p<0.01; # indicates the comparison between the Sal/light compressive stress group and the Sal/heavy compressive stress group, # p<0.05, ## p<0.01).

To explore the effect of eIF-2alpha inhibitor Salubrinal on the mandibular cartilage under compressive mechanical stress, we used H&E staining to observe the thickness of the mandibular cartilage (Fig 1A). The area with degenerative and pathological changes was predominant in the posterior part of the middle third of mandibular cartilage. Compared to the controls, the mandibular cartilage of the light compressive mechanical stress group thinned gradually, and the thickness of the mandibular cartilage reduced to 85% (P<0.05), 70% (P<0.05), and less than 60% (P<0.01) of that of the control group in the 1-, 3-, and 7-day subgroups, respectively (Fig 1F). Degeneration was observed. More lacunas were located in the proliferative zone (Fig 1B, 1-day subgroup of the light compressive mechanical stress group). The chondrocytes in the proliferative zone penetrated into the hypertrophic zones (Fig 1C, 1-day subgroup of the light compressive mechanical stress group).

In the Sal/light compressive mechanical stress group, the thickness of the mandibular cartilage did not increase significantly at day 1 or 3 compared to the light compressive mechanical stress group, but the arrangement of chondrocytes was more organized. In the 7-day subgroup, the thickness recovered to 150% (P<0.05) of that of the light compressive mechanical stress group, and the chondrocytes increased most significantly in the proliferation and hypertrophic zones (Fig 1H).

The mandibular cartilage of the heavy compressive mechanical stress group was even thinner than that of the light compressive mechanical stress group. More progressive degraded changes were observed. The nuclei of the chondrocytes in these degraded areas were typically pyknotic and homogeneous, and the cytoplasm appeared condensed and did not fill the lacuna (Fig 1D, 3-day subgroup of the heavy compressive mechanical stress group). The thickness of the mandibular cartilage reduced to 75% (P<0.05), 65% (P<0.05), and less than 50% (P<0.01) in the 1-day, 3-day and 7-day subgroups, respectively, compared to the control group. The chondrocytes in the mandibular cartilage appeared disordered and disarranged (Fig 1E, 7-day subgroup of the heavy compressive mechanical stress group). The chondrocytes penetrated into the subchondral bone.

Compared to the heavy compressive mechanical stress group, the thickness of cartilage did not increase significantly at day 1 and gradually increased at days 3 and 7 in the Sal/heavy compressive mechanical stress group. The thickness of the mandibular cartilage recovered to 115% (P<0.05) and 138% (P<0.01) of that of the heavy compressive mechanical stress group in the 3-day and 7-day subgroups, respectively. The proliferative and mature zones increased most significantly (Fig 1H). Degeneration of the nuclei and cytoplasm were not observed. The arrangement of chondrocytes was more organized.

Consistent with the thickness of the cartilage, the number chondrocyte cells also decreased gradually in both compressive stress groups and increased in both the Sal/light and Sal/heavy compressive stress groups (Fig 1G).

### 3.3 The effect of Salubrinal on the subchondral bone trabecula under various compressive mechanical stresses

**Fig 2:**
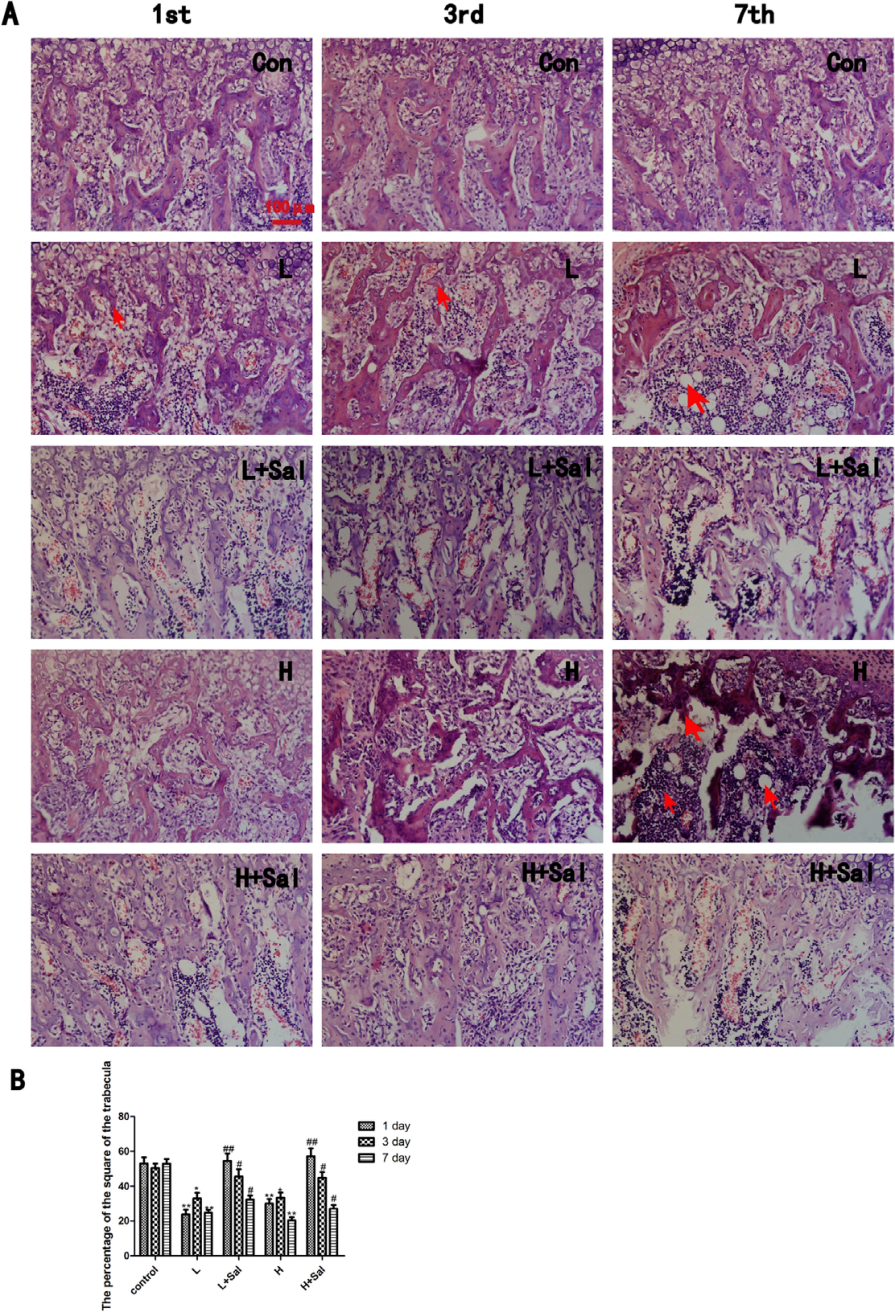
Histological examinations of subchondral bone under various compressive stresses and after injecting Salubrinal. (A) The compressive stresses caused noticeable degenerative changes, including irregular changes to the order and morphology of the bone trabeculae and pathologic changes and inflammation in the heavy compressive stress group. Resorption of the trabeculae is indicated (arrow) in the 1-day and 3-day light compressive subgroups. Vacuole and inflammatory cells are indicated (arrow) in the 7-day compressive subgroup. After the Salubrinal injection, the trabeculae widths recovered. Vacuoles were not observed, and the trabeculae were continuous. In addition, inflammatory cells decreased. (B) The quantitative changes in the percentage of the area of the bone trabeculae. Scale bars, 100 μm. (* indicates the comparison between the compressive stress groups and the control group, * p<0.05, ** p<0.01; # indicates the comparison between the Sal/compressive stress groups and the compressive stress groups, # p<0.05 ## p<0.01).

We examined whether different compressive mechanical stresses caused different changes in the architecture of the mandibular subchondral bone. H&E staining revealed obvious degenerative changes, including irregular changes to the order and morphology of the bone trabeculae and pathologic changes in the compressive mechanical stress groups (Fig 2A).

In the light compressive mechanical stress group, resorption of the trabeculae occurred horizontally and vertically. In the 1-day and 3-day subgroups, the trabeculae became thin, and the intertrabecular space became wider as a result (Fig 2A). In the 7-day subgroup, vacuole and inflammatory cells were observed, and the trabeculae became discontinuous. Inflammatory cells and micrangiums increased between trabeculae. After the Salubrinal injection, the width of the trabeculae recovered, particularly in the 7-day subgroup. Vacuoles were not observed, and the trabeculae were continuous. In addition, inflammatory cells decreased, although dilated micrangiums remained between the trabeculae.

In the heavy compressive mechanical stress group, the trabeculae resorbed extensively. The pathologic changes were most significant in the 7-day subgroup and took the form of fractures, vacuole and inflammatory cell infiltration. After the Salubrinal injection, the disorder of the bone trabeculae abated and recovered. Continuous trabeculae and alleviated inflammation could be observed. Fractures and vacuole disappeared.

The percentage of the area of the bone trabeculae in both compressive mechanical stress groups decreased to approximately 50% (P<0.01) of the control group after pressure loading at day 1 (Fig 2B). In the 3-day subgroup, the area recovered to approximately 65% (P<0.05) of the control group and then decreased to less than 50% (P<0.01) of the control group in the 7-day subgroup. There was no significant difference between the light and heavy compressive mechanical stress groups. After the Salubrinal injection, the percentage of the area of the bone trabeculae in both groups recovered to almost the same level as the control group at day 1. In the 3-day subgroup, the area recovered to approximately 136% (P<0.05) of that of the compressive mechanical stress groups. In the 7-day subgroup, the area was approximately 133% (P<0.05) of that of the compressive mechanical stress group. There was no significant difference between the Sal/light and Sal/heavy compressive mechanical stress groups.

### 3.4 Ultrastructure observation

**Fig 3:**
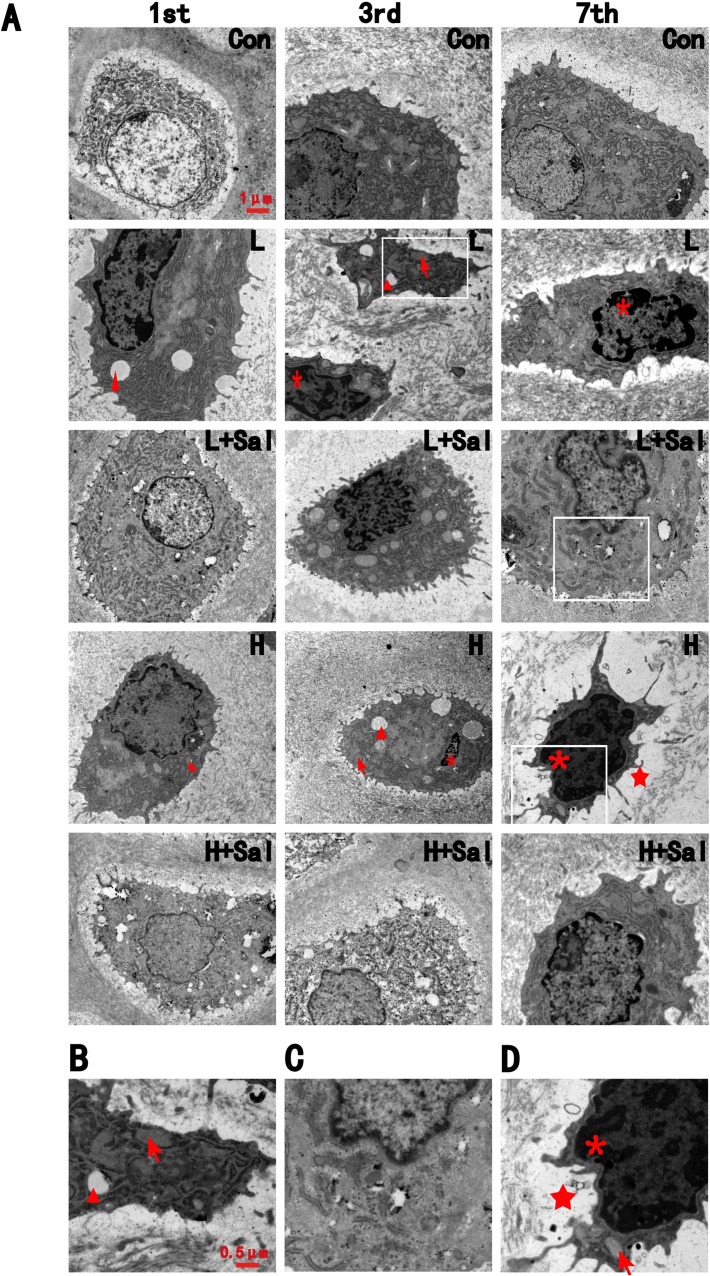
TEM revealed the ultrastructure change of chondrocytes. (A) The ultrastructure change of chondrocytes under various compressive stresses after injecting Salubrinal at days 1, 3 and 7.ER dilation is indicated by an arrow, condensed chromatin is indicated by an asterisk, vesicles are indicated by triangles, and decreasing collagen deposition indicated by a pentagram. Scale bars, 1 μm. (B) Higher magnification in the day-3 light compressive stress subgroup. ER dilation is indicated by an arrow, vesicles are indicated by triangle. Scale bars, 0.5 μm. (C) Higher magnification in the day-7 light compressive stress subgroup after injecting Salubrinal. (D) Higher magnification in the day-7 heavy compressive stress subgroup. ER dilation is indicated by an arrow, condensed chromatin is indicated by an asterisk,and decreasing collagen deposition indicated by a pentagram.

In the control group, most chondrocytes were located in the lacunas and contained few vesicles in the cytoplasm. The region around the nuclei of the cells showed well-developed mitochondrial cristae and endoplasmic reticulum (Fig 3A).

In the light compressive mechanical stress group, vesicles and dilation of endoplasmic reticulum in the cytoplasm were observed after 1 day (Fig 3B). In the 3-day and 7-day subgroups, condensed and irregular nuclei could be observed. After the Salubrinal injection, dilation of the endoplasmic reticulum in the cytoplasm could still be observed in the 1-day and 3-day subgroups, but vesicles in the cytoplasm decreased. In the 7-day subgroup, cell degeneration recovered. The dilation of the endoplasmic reticulum was repressed, and condensed nuclei could not be observed (Fig 3C).

In the heavy compressive mechanical stress group, vesicles and dilation of endoplasmic reticulum were observed at days 1 and 3. These were accompanied by small patches of condensed chromatin spreading throughout the nuclei. At day 7, the nuclei became condensed and irregular and had more incisures (Fig 3D). Moreover, collagen deposition around the chondrocytes in the 7-day subgroup decreased significantly (Fig 3D).

In the Sal/heavy compressive stress group, there were still vesicles and expansion of the endoplasmic reticulum in the cytoplasm at day 1. Ovoid nuclei containing granular chromatin material could be observed after 3 days. In contrast to the compressive mechanical stress group, cell degeneration recovered after 7 days in the Sal/heavy compressive stress group, although there are still patches of condensed chromatin in the nuclei. Collagen deposition around the chondrocytes in the 7-day subgroup also recovered.

### 3.5 Proliferation and apoptosis of chondrocytes under various compressive mechanical stresses after injecting Salubrinal

**Fig 4:**
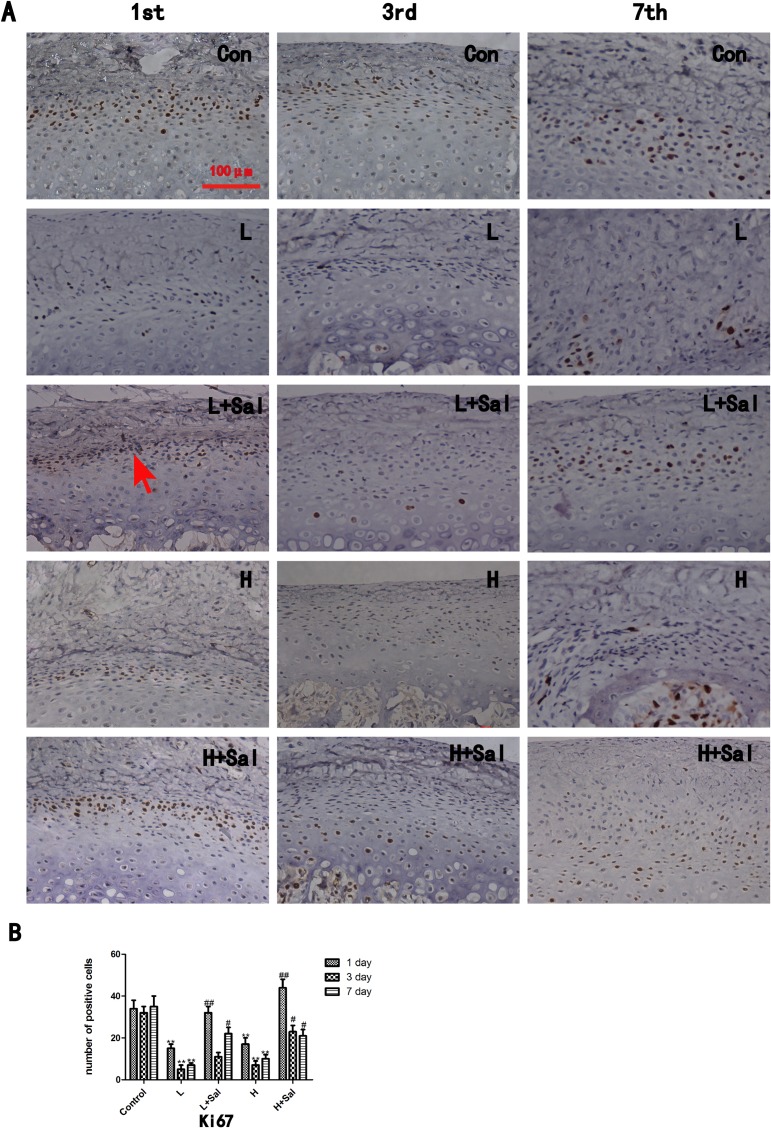
Ki67 immunostaining. **(A)**Ki67 immunostaining. A Ki67 positive chondrocyte is indicated (arrow). Scale bars, 100 μm. (B)The Ki67 positive cells were counted. (* indicates the comparison between the compressive stress groups and the control group, * p<0.05, ** p<0.01; # indicates the comparison between the Sal/compressive stress groups and the compressive stress groups, # p<0.05, ## p<0.01)Apoptosis was examined using a TUNEL assay (Fig 5A and 5B). In the control group, few apoptotic cells were detected in the mandibular cartilage. In the light compressive mechanical stress group, the number of apoptotic cells increased significantly at day 1 (P<0.01) and then gradually decreased at days 3 and 7. After the Salubrinal injection, the number of apoptotic cells decreased at day 1 (P<0.01) compared to the light compressive mechanical stress group, and few apoptotic cells could be detected at days 3 and 7. The number of apoptotic cells in the heavy compressive mechanical stress group increased gradually from day 1 (P>0.05) to day 3 (P<0.05) and was still high in the 7-day subgroup (P<0.01) compared to the control group. By contrast, the number of apoptotic cells was much lower in the Sal/heavy compressive stress group than in the heavy compressive stress group. In the Sal/heavy compressive mechanical stress group, the number of apoptotic cells decreased slightly at day 3 (P<0.05) and then decreased significantly at day 7 (P<0.01) compared to the heavy compressive mechanical stress group.

In comparison to the control group, the expression of proliferative protein Ki67 in both the light and heavy compressive stress groups decreased significantly until day 3 (P<0.01) and then recovered slightly (Fig 4A and 4B). There was no significant difference in Ki67 expression between the light and heavy compressive stress groups. Compared to the light compressive stress group, Ki67 expression in the Sal/light compressive stress group increased significantly at day 1 (P<0.01) and day 7 (P<0.05). In the 3-day subgroup, Ki67 expression increased slightly compared to the light compressive stress group. In the Sal/heavy compressive stress group, Ki67 expression increased most significantly at day 1 (P<0.01) compared to the heavy compressive stress group. Although it decreased over time, Ki67 expression was still higher than that of the heavy compressive stress group at days 3 and 7 (P<0.05).

**Fig 5:**
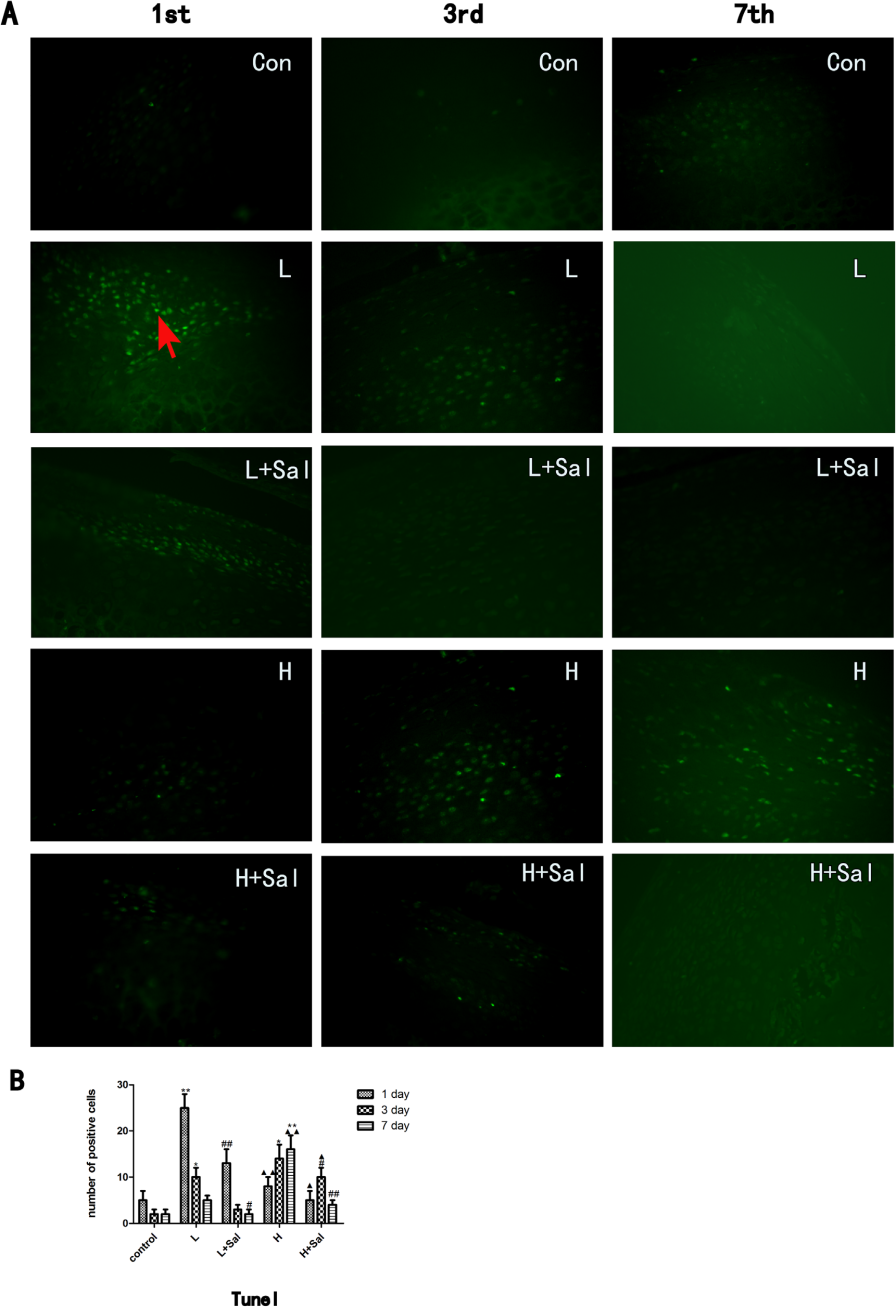
TUNEL assay. (A) TUNEL assay. A TUNEL positive chondrocyte is indicated (arrow). (B) The TUNEL positive cells were counted. (* indicates the comparison between the compressive stress groups and the control group, * p<0.05, ** p<0.01; ▲ indicates the comparison between the light and heavy compressive stress groups, ▲ p<0.05, ▲▲ p<0.01; # indicates the comparison between the Sal/compressive stress groups and the compressive stress groups, # p<0.05, ## p<0.01).

## Discussion

The cartilage thickness and number of cells decreased gradually in both the light and heavy compressive stress groups, and the cartilage thickness was noticeably thinner in the heavy compressive stress group. However, no significant differences in the percentage of the area of subchondral bone trabeculae were found between the two groups. Our results indicate that changes in cartilage thickness were closely related to the force value, but changes in the subchondral bone were not. Cartilage is a stress-distributing and load-absorbing structure, while subchondral bone is an underlying rigid-bony bed that serves as cartilage support [13]. Moreover, cartilage contains only type of one cell and has no vascular, lymphatic or neural supply [14]. Therefore, cartilage responded directly and subchondral bone responded indirectly to mechanical stimulation. Chondrocytes reduce in number and alter their distribution, leading to diminished maintenance and repair. In addition, more pathological changes, such as fractures, bone cysts and inflammation, were found in the heavy compressive stress groups, indicating that the subchondral bone was more fragile and could break more quickly under excess mechanical stress [1,15].

The proliferation changes were in line with the histological changes of the mandibular cartilage and also related to the force magnitude. Decreasing metabolic activity and reduced proliferation of mandibular chondrocytes contributed the thinning of the cartilage. The apoptosis changes differed between the heavy and light compressive stress groups. The light force induced apoptosis in the early loading stage (day 1) and then dropped very quickly. The heavy force induced apoptosis gradually with the loading time, reaching its highest level in the 7-day subgroup but not as significantly as did the light force group at day 1. All results indicate that the inhibition of proliferation occurred during all loading times. In addition, light force triggered the early activation of apoptosis, while heavy force triggered its late activation.

As an agent that could elevate eIF2α, Salubrinal's advantage is that it has a potential therapeutic possibility for inflammatory arthritis [16]. Many studies have shown multiple beneficial protecting effects of Salubrinal on skeletal tissue, and our results supported these findings. We have also demonstrated its chemoprotective effect on cartilage. Salubrinal can promote the proliferation of chondrocytes, significantly decrease apoptosis, and protect subchondral bone and ultrastructures under light and heavy compressive stresses at all loading times, all of which contributed to the recovery of the thickness of the cartilage and of the pathological changes in the subchondral bone in both groups. Previous studies have shown that Salubrinal could abolish the suppression of cell proliferation [17] and also decrease apoptosis by increasing eIF2α phosphorylation [18], which is in line with our results. Salubrinal also had timing effects in the present study. It altered the proliferation and apoptosis of chondrocytes most significantly in the early loading stage (day 1) in the light force group. Under the heavy compressive stress stimulation, Salubrinal increased the proliferation of chondrocytes at day 1 and reduced the apoptosis of chondrocytes most significantly at day 7 (the late loading stage). Furthermore, pathological changes of the subchondral bone, such as fractures, vacuole, fibrosis and inflammatory cell infiltration, in the heavy compressive stress group were alleviated after injecting Salubrinal, which indicates that this drug could suppress inflammatory responses in bones [19]. In addition, the percentage of the area of bone trabeculae in the compressive stress groups recovered after Salubrinal was injected, which indicates that Salubrinal plays a role in the stimulation of bone formation [9].

## Conclusion

In summary, we developed various compressive stress rat models. Degeneration, pathological changes and inflammation could be observed in cartilage and subchondral bone. The cartilage thickness and number of cells gradually decreased in both the light and heavy compressive stress groups, and these changes were related to force magnitude and loading time. After the Salubrinal injection, the thickness of the cartilage recovered, and the pathological changes were alleviated. In the Sal/light compressive stress group, the drug altered the proliferation and apoptosis of chondrocytes most significantly at day 1. In the Sal/heavy compressive stress group, the drug increased the proliferation of chondrocytes most significantly at day 1 and reduced the apoptosis of chondrocytes most significantly at day 7. In addition, Salubrinal increased the area of the bone trabeculae and suppressed inflammatory responses and pathological change in the subchondral bone of the TMJ.

## Supporting Information

S1 Fig Overall view of mandibular condylar cartilage. The squares located in the middle third of the cartilage and the subchondral bone are the main load-bearing areas based on the direction of force application.
(TIF)

S2 Fig Fold change of the thickness of cartilage, area of subchondral bone, Ki67 positive chondrocytes, and TUNEL positive chondrocytes. (A) Fold change in the thickness of the cartilage. The measured value in the control group was set as 1. (B) Fold change of the area of subchondral bone. (C) Fold change of Ki67 positive chondrocytes. (D) Fold change of TUNEL positive chondrocytes.
(TIF)
