# Supplementary figures and images for: The Protective Effects of Salubrinal on the Cartilage and Subchondral Bone of the Temporomandibular Joint under Various Compressive Mechanical Stimulations
Source: PLoS One. 2016 May 19;11(5):e0155514. doi: 10.1371/journal.pone.0155514 (PMC4873203; doi:10.1371/journal.pone.0155514)

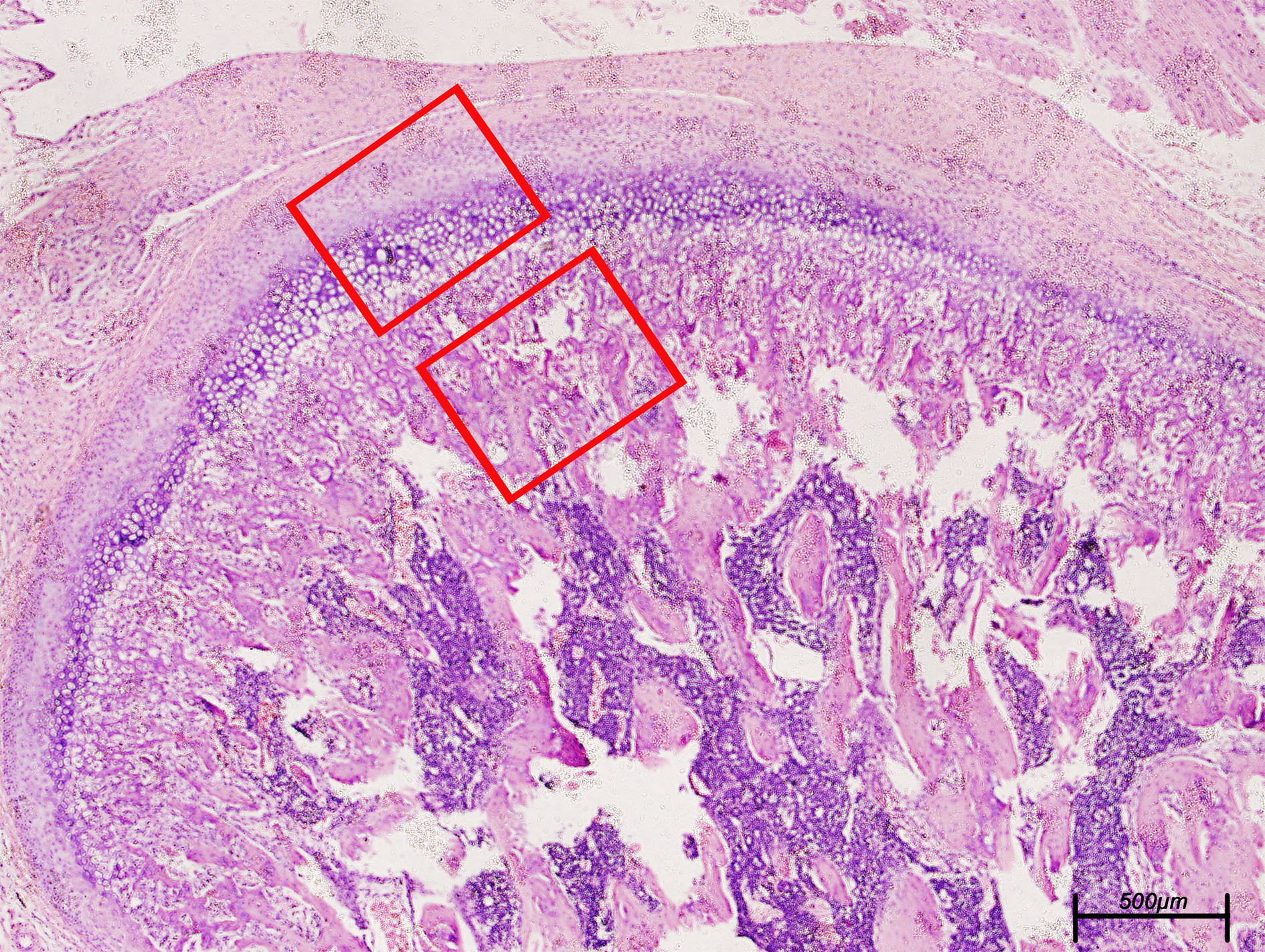

Supplement: S1 Fig — The squares located in the middle third of the cartilage and the subchondral bone are the main load-bearing areas based on the direction of force application. (TIF) [file pone.0155514.s001.tif]

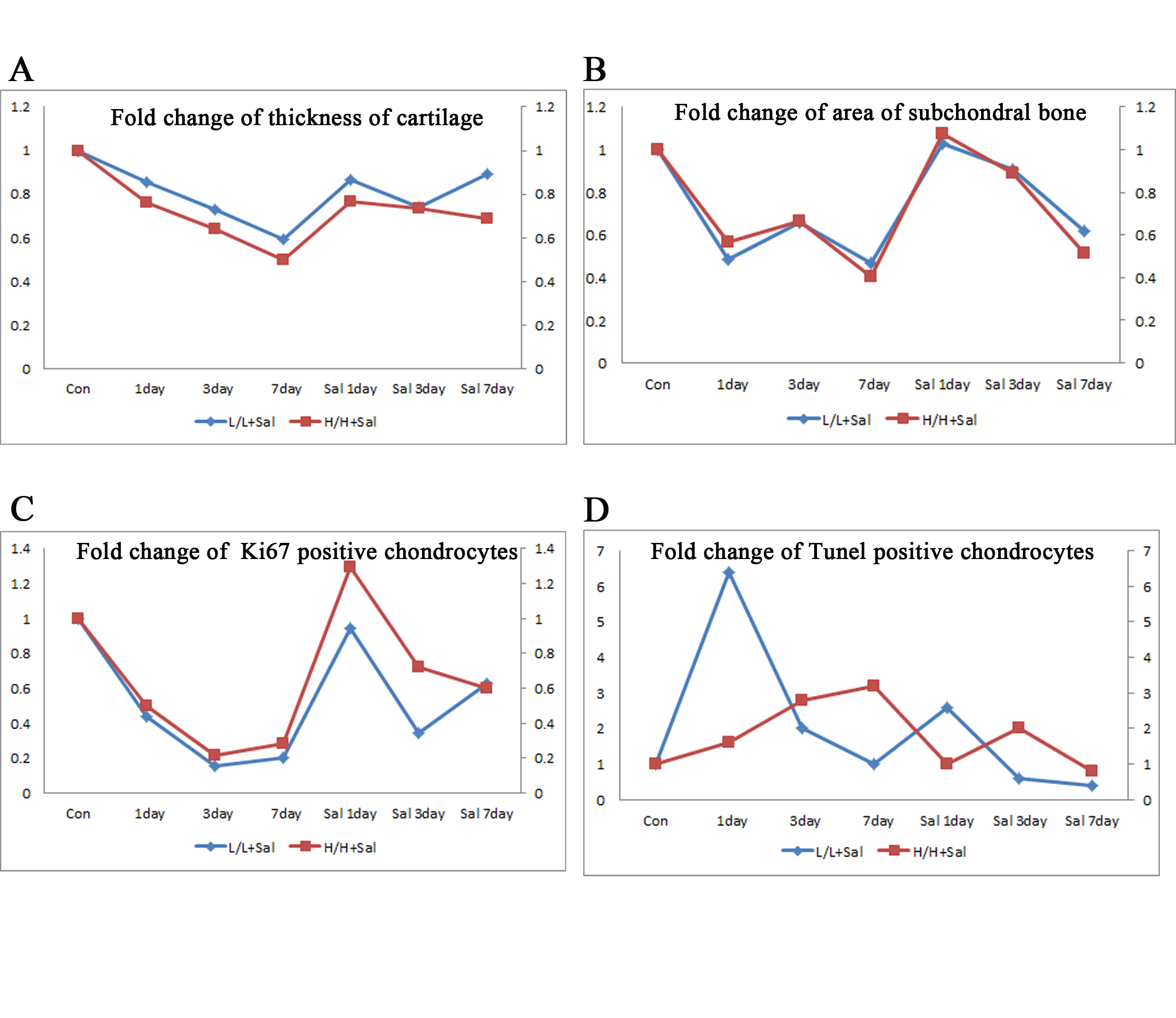

Supplement: S2 Fig — (A) Fold change in the thickness of the cartilage. The measured value in the control group was set as 1. (B) Fold change of the area of subchondral bone. (C) Fold change of Ki67 positive chondrocytes. (D) Fold change of TUNEL positive chondrocytes. (TIF) [file pone.0155514.s002.tif]
